# Prostitution use has non sexual functions - case report of a depressed psychiatric out-patient

**DOI:** 10.12688/f1000research.2-70.v2

**Published:** 2013-05-29

**Authors:** Fátima Gysin, François Gysin

**Affiliations:** 1 F+F Gysin, Private Psychiatric Practice, Campo Grande 156 -2º, 1700-094 Lisbon, Portugal

**Keywords:** paid sex use, prostitution, depression, failure of genital response, erectile disorder, girlfriend-experience, sex worker, sex-work-model, oppression-model, Arizona Sexual Experience Scale

## Abstract

**Case:** A shy, depressed 30 year old male discussed his frequent ego-syntonic indoor prostitution consumption in small peer groups. Several distinctive non-sexual functions of this paid sex habit were identified.

**Design and method: **The patient had 40 hourly psychiatric sessions in the private practice setting over 14 months. The Arizona Sexual Experience Scale was applied to compare the subjective appraisal of both paid sex and sex in a relationship. The informal Social Atom elucidates social preferences and the Operationalized Psychodynamic Diagnostic-procedure was applied to describe a dominant relationship pattern.

**Results:** The paid sex consumption functioned as a proud male life style choice to reinforce the patients fragile identity. The effect on self esteem was a release similar to his favorite past-time of kick-boxing. With paid sex asserted as a group ritual, it was practiced even with frequent erectile dysfunction and when sex with a stable romantic partner was more enjoyable and satisfying. The therapeutic attitude of the female psychiatrist, with her own ethical values, is put in to context with two opposing theories about prostitution: the ‘Sex-Work-model’ and the ‘Oppression-model’. The therapist’s reaction to the patients’ information was seen as a starting point to understanding the intrapsychic function of paid sex as a coping mechanism against depressive feelings.

**Conclusions: **Exploring and understanding prostitution consumption patterns in young men can benefit the treatment of psychiatric disorders in the private practice setting. It is the psychiatrists task to investigate the patients hidden motives behind paid sex use to help patients achieve a greater inner and relational freedom.

## Introduction

Paid sex behavior, either giving or receiving money, is a delicate matter for psychiatric patients. Patients rarely talk about this taboo subject spontaneously, and habitually psychiatrists tend not to ask about it. On the other hand, prostitution use is frequent; 18% of American men paid for sex in their past, and 3% of them did so during the year before inquiry ^1^. 2% of American women have received money for sex during their lifetime ^1^.

We hypothesize that the personal and therapeutic attitude of health professionals towards paid sex is often ambivalent leading towards the avoidance of the topic. There are multiple reasons for this. We hypothesize before the background of our education and practice in Switzerland and Portugal the following possible reasons: 1. Reduced importance of the topic in medical education and in psychiatry-training. 2. A certain degree of idealizing the patient as not being prone to morally questionable behavior. 3. Absence of apparent clues in the patient’s presentation and in his narrative.

We think that particularly in young men, asking actively about and understanding prostitution consumption may benefit their psychiatric and psychotherapeutic treatment. In our experience, investigating a patient’s hidden motives behind paying for sex can help patients to achieve greater inner and relational freedom.

## Case

### The patient

Mr. A, 30 years old, small and shy, was born in a north-western Portuguese village near an internationally renowned Casino-beach-resort and lived there until the age of 18. He is the only son of a working-class couple, both in employment. Mr. A attended a college in Lisbon, which was a three hour drive from his parent’s home. None of his peers from the village went to college. Mr. A was a driven individual and achieved his goal of pursuing further education.

At home his parents “always quarreled” about his father’s infidelities but they stayed together to finance his studies. When they divorced after his graduation, he felt sad and developed gradually a depressed mood. His symptoms lasted for one year before he came for a consultation to the first author Fátima Gysin.

Additionally, he broke two toes kick-boxing, causing him to stop practicing his favorite sport. During this period he also started to date a girl who had a poor education and no fixed job. She envied him for his higher income and she was unfaithful to him. For these reasons and with a noticeable low performance in his engineering job, he was encouraged by his friends and pressured by his superiors at work to see a psychiatrist, whom he selected from a health insurance list.

During his first sessions in September 2011 he showed low mood, he was resentful for not being promoted at work, had lack of motivation, social isolation and criticized his bizarre feelings when he was irrationally afraid of being attacked in a familiar and secure night club. He had outbursts with friends, anhedonia, a lesser sexual drive, fear of losing his hair and of gaining weight against the evidence and he was excessively dysphoric of his small height of 1.59m. He wanted help but without medication.

### He pays for sex

For Mr. A, paid sex was not a problematic issue, nor a direct motive for his consultation. When questioned about his sex-life he was comfortable talking about his experiences, and showed a consumer pride in prostitution. At the age of 22, after breaking up a four-year relationship, out of curiosity and revenge, he purchased for the first time sexual services in a sex-worker’s apartment. This use of prostitution escalated when back in his native village, where a Saturday night ritual with a group of friends started. After dinner they would bring home their official girlfriends and then, only the four or five boys, visit a brothel, have a drink, have fun and sometimes have paid sex with prostitutes, using protection. This kind of prostitution use became a peer group standard and, for Mr. A, an easy victory over his shyness.

Mr. A became hooked and hyper-seduced ^2^ by a specific prostitute, and he fantasized about living with her in an exotic land. However, to have or to maintain a full erection during paid sex he needed to think and imagine that he was making love to a romantic partner. The patient referred to exclusive heterosexual orientation and sexual desire, however revealed that he would suffer a loss of erection when either nervous or stressed.

## Design, methods and results

### Tests and scales

The Arizona Sexual Experience Scale (ASEX) is designed to assess five major global aspects of sexual dysfunction: drive, arousal, penile erection/vaginal lubrication, ability to reach orgasm, and satisfaction from orgasm ^3^. ASEX can be used regardless of the availability of a sexual partner, and the questions are short, easy to understand, and less intrusive than questions typically found in more traditional tools. It is an effective tool at accurately measuring sexual dysfunction if present ^3^.

**Figure 1.**
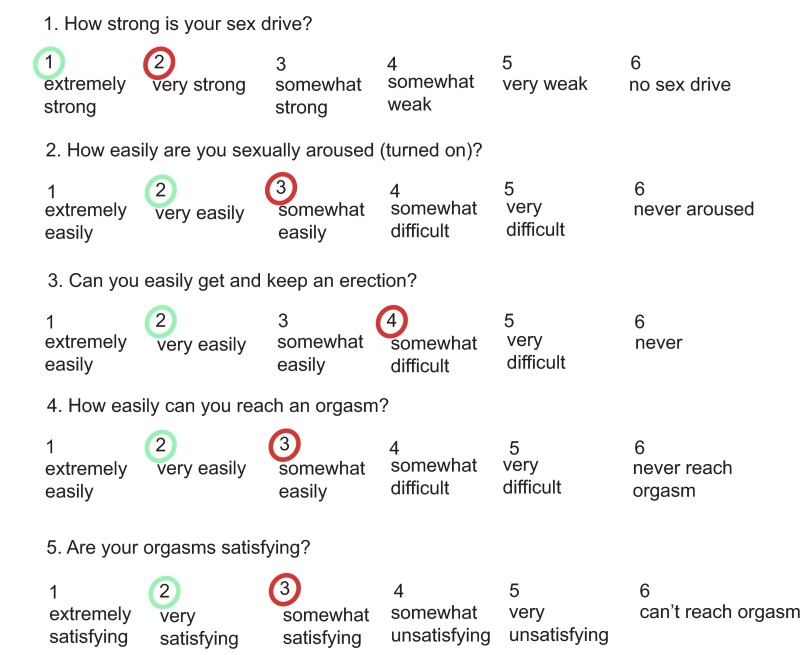
Mr. A’s Arizona Sexual Experience Scale revealing that paid sex is less intense compared with romantic sex.

As we can see in Figure 1, for Mr. A, paid sex (red circles) is less exciting and less intense compared to romantic sex (green circles). There is a difference of one number on the Likert-scale of the ASEX for all the questions asked, except for the “can you get and keep an erection?” question where the divergence is two numbers. This divergence indicates Mr. A suffers from some degree of erectile dysfunction with regards to paid sex. The question arises why Mr. A continued to seek paid sex whilst he was having what he said was more desirable romantic sex with his partner.

**Figure 2.**
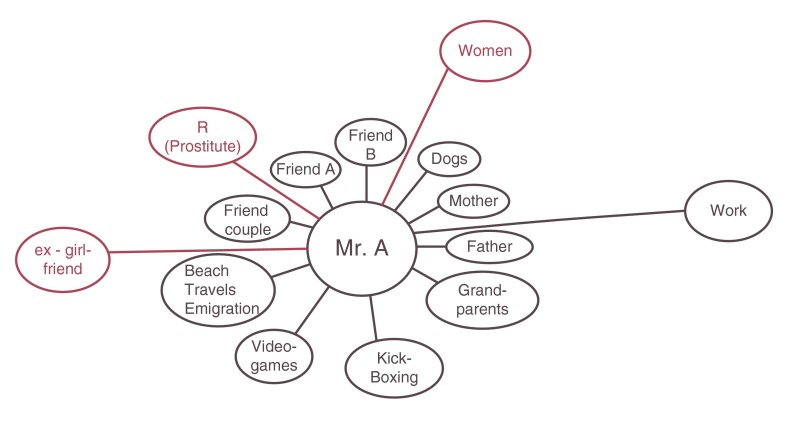
Mr. A’s Social Atom shows problematic affective intimacy to women.

The informal Social Atom ^4^ is a drawing that the patient was asked to create, drawing circles to represent his most important relationships and favorite past-times (see Figure 2). The proximity to the subject (the middle circle) indicates the importance of these to him. Mr. A’s social atom showed close relationships to his parents, friends and pets and also confirmed his problematic affective intimacy with women.

### Mr. A’s relationship-cycle

We can describe Mr. A’s relationship dynamics by the means of anamnestic data and through his behavior in the therapeutic setting. We followed the Operationalized Psychodynamic Diagnostic-procedure ^5^.

**Figure 3.**
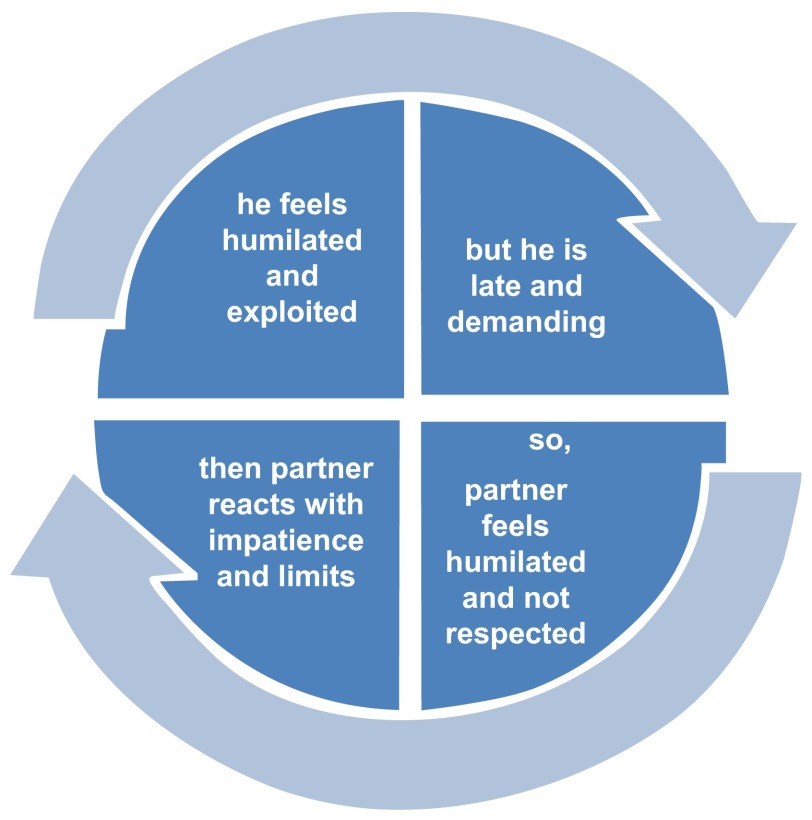
Mr. A’s dynamic relationship cycle – this maladaptive repetitive pattern of feelings and behaviors is mostly non conscious to Mr. A.

Mr. A felt that he was not being recognized in his efforts to fulfill or please others. Often arriving late to dates with girlfriends, he not only refused to apologize, but also expected them to listen supportively to his complaints. He implicitly asked too much of others without being aware of it, and then found himself surprised by the negative response he received, and found this negativity unjustified and rejecting. He felt an imbalance between giving and receiving, which reinforced his fears of intimacy (see Figure 3).

With his paid sex encounters, he tried to escape from this cycle. After sex for money, he felt, nobody is in debt with anyone. He looked for a “girlfriend experience” free from any affective claims. His internalized couple model is characterized by infidelity, hostility and matrimonial warfare.

For Mr. A, paid sex had many simultaneous non-sexual functions, following Willy Pasini’s list of Non-sexual Functions of Sex ^6^: sex as tranquilizer and antidepressive for his symptoms, as identity-support and socializer in his peer group as well as power-tool and object of exchange with women. The intrapsychic function of paid sex seems to be a narcissistic first-aid kit.

For conscious and expressed reasons of why people pay for sex we can refer to a recent taxonomy of motifs for sex ^7^, which consists of four large factors and 13 sub-factors: **1. “Physical Reasons”** which includes *stress reduction*, *pleasure*, *physical desirability*, and *experience seeking*. **2. “Goal Attainment”** which includes *resources*, *social status*, *revenge*, and *utilitarian*. **3. “Emotional”** which includes *love and commitment*, and *expression*. **4. “Insecurity”** which includes *self-esteem boost*, *duty/pressure*, and *mate guarding*. The paid sex experiences of Mr. A may have satisfied at least seven of these motifs: *stress reduction, pleasure, physical desirability, experience seeking, social status, revenge* and *self-esteem boost*.

### Diagnosis and clinical evolution

Mr. A was diagnosed with depressive episode, moderate to severe, with mild psychotic symptoms (ICD-10: F33.2, corresponding to DSM-IV-TR: 296.24) ^8, 9^ and failure of genital response (episodic erectile dysfunction in a paid sex setting) (ICD-10: F52.2, corresponding to DSM-IV-TR: 302.72). Paid sex activity in general may hide the aspect of a disorder of impulse control ^10^, however this was not present with Mr. A. Addictive and obsessive traits in his paid sex behavior were ruled out. A weekly psychotherapeutic treatment was proposed and started in monotherapy, without antidepressive medication. The patient showed high therapy-motivation and good compliance. He accepted the therapy-program and during a period of 14 months, Mr. A attended 40 hourly sessions.

The patient gradually improved without psychotropic medication and took three weeks off work as sick-leave and three weeks off work for overdue vacations. He quickly changed to a more challenging and better rewarded job and started up kick-boxing again. Socializing better in his new job, he continued solitary in his private life and started to commit time to his newly adopted dog. Since caring for dogs was his mother’s favorite activity, this newly found past time suggests possible feelings of closeness towards her and also allows him to identify with the more enjoyable aspects of his upbringing.

Moreover, he reduced his peer-group paid sexual activities but still dissipated his energy by his regular night-life, drinking and paid sex consumption. His sentimental life still revolved around his problematic girlfriend, whom he had chosen when he was depressed.

## Discussion

### Theories of prostitution

To understand the patient’s pattern of paid sex consumption and the therapeutic attitude and reaction of the therapist, it is important to understand the actual positions and knowledge about prostitution. There is general agreement that one should distinguish clearly between street and indoor prostitution ^11^. Street prostitution is seen as implying frequent health-risks for workers with a high degree of victimization and oppression, while indoor prostitution is a subject of much debate.

Two main prostitution theories explain and deal with indoor prostitution: the “Sex-Work model” ^12^ and the “Oppression model” ^13^. The first proposes legalization of prostitution as a way to earn a living, and for harm reduction, minimizing risks for sex workers and consumers. The second focuses on exploitation, victimization and abuse of women and is usually adopted by feminist movements ^13^ and right-wing conservatives for different reasons ^12^.

Portugal does not criminalize prostitution, but linked economic activities are illegal, like renting an apartment for prostitution work. There is no regulation, and sex workers are without legal protection and do not benefit from systematic harm reduction strategies. In general, prostitution does not seem to be on sexological or political agendas and very few studies about prostitution-users are available ^14^.

### Considerations on Mr. A’s therapy and debate

Although the patient suffered from a moderate to severe depressive episode, with mild psychotic signs, no medication was given in response to the patients’ wishes and following the tendency of psychiatrists to follow a “watchful waiting” approach. Official therapeutic recommendations in Germany and Switzerland state: “When a unique therapeutic approach is planned in patients suffering from moderate to severe acute depressive episodes, treatable in outdoor-setting, exclusive psychotherapy should have the same importance as exclusive medication when the method of treatment is chosen ^15^”.

The attitudes of psychiatrists and psychotherapists to paid sex are rarely examined. With the clinician, a professional tolerant and neutral position may coexist with a private negative sensibility about prostitution. Unintentionally, this may result in a negative judgment of paid sex and avoidance of the patient’s narratives of paid sex behavior.

Even the most comprehensive and recent “Standard Operating Procedures for Taking a Sexual History” ^16^ doesn’t refer at all to paid sex experiences. Their very practical “prospective opening questions”, “follow-up questions” and “more specific questions” focus on sex within relationships and do not give the clinician any tools to help the patient talk and listen openly about their paid sex experiences.

The first author (therapist of Mr. A) specialized in sexology in a sexological and psychosomatic consultation in the University of Geneva headed by Willy Pasini. In Portugal, in the private practice setting, she has treated an up-scale sex-worker for depression and anxiety unrelated to her professional activity. The patient valued her work and was determined to continue indoor, top-level prostitution. In this context, exploitation, misery and victimization are absent or less visible and sex-work appears to be frequently a free choice of profession.

One question is the degree of communication of the therapist’s personal moral, ethical or political stance to the patient. Sometimes the therapeutic process is better served by the therapist’s auto-disclosure, while generally non-disclosure is recommended ^17^.

## Conclusion

Although it wasn’t a reason for consultation nor was it presented as a symptom, it was essential to Mr. A’s psychiatric treatment for his depressive disorder to open up his paid sex habits. The therapy helped him to make sense of and give meaning to his struggle with intimacy. The change from his depressive mood was probably facilitated by the interest given to the significance of his paid sex experience. On different levels, prostitution use can be simultaneously a symptom, a free choice and a cultural pattern. In depressed men seeking psychiatric or psychotherapeutic help, an active exploration of any existing paid sex experiences can be useful.

## Consent

Written informed consent for publication of the clinical details was obtained from the patient. Permission has been solicited by e-mail for publishing the results of the Arizona Sexual Experience Scale (ASEX).
