# Supplementary material for: Prostitution use has non sexual functions - case report of a depressed psychiatric out-patient
Source: F1000Res. 2013 May 29;2:70. Originally published 2013 Mar 4. [Version 2] doi: 10.12688/f1000research.2-70.v2 (PMC3924946; doi:10.12688/f1000research.2-70.v2)
Supplement: Peer review history

# Peer review: Referee response for version 2

**DOI:** 10.5256/f1000research.1524.r4140

**Published:** 2014-03-17

**Authors:** Antonio Pacheco Palha

**Affiliations:** 1 Department of Psychiatry, Hospital San Joao, University of Porto, Porto, Portugal

I have read this submission. I believe that I have an appropriate level of expertise to confirm that it is of an acceptable scientific standard.

# Peer review: Referee response for version 2

**DOI:** 10.5256/f1000research.1524.r980

**Published:** 2013-06-03

**Authors:** Chiara Simonelli, Marta Giuliani, Stefano Eleuteri

**Affiliations:** 1 Dipartimento di Psicologia, Istituto di Sessuologia Clinica, Rome, Italy; 2 Institute of Clinical Sexology, Rome, Italy; 3 “Sapienza”, University of Rome, Rome, Italy

The manuscript is much improved.

We have read this submission. We believe that we have an appropriate level of expertise to confirm that it is of an acceptable scientific standard.

# Peer review: Referee response for version 1

**DOI:** 10.5256/f1000research.857.r897

**Published:** 2013-04-17

**Authors:** Antonio Pacheco Palha

**Affiliations:** 1 Department of Psychiatry, Hospital San Joao, University of Porto, Porto, Portugal

It is only one clinical case and so I think it has a medium interest. Although it is present in a clear way I think the discussion should be more consistent. Anyway, it deserves to be published.

I have read this submission. I believe that I have an appropriate level of expertise to confirm that it is of an acceptable scientific standard.

# Peer review: Referee response for version 1

**DOI:** 10.5256/f1000research.857.r828

**Published:** 2013-03-11

**Authors:** Chiara Simonelli, Marta Giuliani, Stefano Eleuteri

**Affiliations:** 1 Dipartimento di Psicologia, Istituto di Sessuologia Clinica, Rome, Italy; 2 Institute of Clinical Sexology, Rome, Italy; 3 “Sapienza”, University of Rome, Rome, Italy

This is a complete case report, where all the necessary information has been extensively provided. Overall, the report is valuable, but we have few reservations. We suggest to the Authors to:

- Explain more clearly some of the clinical considerations in order to facilitate the reader’s comprehension of this case. For example, they could better specify the sentence; 'which coincidentally was his mother’s favorite activity', on page 4;
- Within the text, if possible, comply with paragraphs’ name used in the abstract;
- Add in the abstract (section: Design and method) the informal Social Atom and the Operationalized Psychodynamic Diagnostic-procedure;
- Specify in the section 'Tests and scales' the important results emerging from Arizona Sexual Experience Scale, as it has been done for the Social Atom;
- Add to the ICD-10 nosography also the DSM classification: in some Countries this is more used than the other one and so it could be more comprehensible;
- The reference list could be improved with some recent international articles supporting your theoretical assumptions. For example we can suggest two important publications concerning different motivations to sexuality and the importance to talk about sexuality during the therapeutic process:   
- Meston CM, Buss DM (2007), Why Humans Have Sex, *Archives of Sexual Behaviour*, **36**: 477-507
- Althof SE, Rosen RC, Perelman MA, Rubio-Aurioles E (2013), Standard operating procedures for taking a sexual history, *Journal of Sexual Medicine*, **10**: 26-35.

We have read this submission. We believe that we have an appropriate level of expertise to confirm that it is of an acceptable scientific standard, however we have significant reservations, as outlined above.
